# Design of a Novel MEMS Gyroscope Array

**DOI:** 10.3390/s130201651

**Published:** 2013-01-28

**Authors:** Wei Wang, Xiaoyong Lv, Feng Sun

**Affiliations:** College of Automation, Harbin Engineering University, No. 145 Nantong Street, Harbin 150001,Heilongjiang, China; E-Mails: lengfeng0506@163.com (X.L.); Sunfeng_hrbeu@163.com (F.S.)

**Keywords:** structural design, MEMS, gyroscope array, multi-DOF, wide bandwidth

## Abstract

This paper reports a novel four degree-of-freedom (DOF) MEMS vibratory gyroscope. A MEMS gyroscope array is then presented using the novel gyroscope unit. In the design of the proposed 4-DOF MEMS vibratory gyroscope, the elements of the drive-mode are set inside the whole gyroscope architecture, and the elements of sense-mode are set around the drive-mode, which thus makes it possible to combine several gyroscope units into a gyroscope array through sense-modes of all the units. The complete 2-DOFvibratory structure is utilized in both the drive-mode and sense-mode of the gyroscope unit, thereby providing the desired bandwidth and inherent robustness. The gyroscope array combines several gyroscope units by using the unique detection mass, which will increase the gain of sense-mode and improve the sensitivity of the system. The simulation results demonstrate that, compared to a single gyroscope unit, the gain of gyroscope array (*n* = 6)is increased by about 8 dB; a 3 dB bandwidth of 100 Hz in sense-mode and 190 Hz in drive-mode are also provided. The bandwidths of both modes are highly matched with each other, providing a bandwidth of 100 Hz for the entire system, thus illustrating that it could satisfy the requirements in practical applications.

## 1. Introduction

Since the first micro machined tuning-fork gyroscope was reported by Draper laboratory in 1991 [1],many types of MEMS gyroscopes have been designed and manufactured. Due to advantages such as small size, low cost, reduced power consumption, and so on, micro machined vibratory gyroscopes have received a great deal of attention in the past several decades. Currently, some micro machined vibratory gyroscopes have been utilized in a variety of practical applications, such as in the automotive industry, electronics, and INS(inertial navigation system) for tactical weapons and vehicles, *etc*.

Conventional micro machined vibratory gyroscopes mainly employ the architecture with 1-DOFdrive-mode and sense-mode, which increases the mechanical gain by matching the natural frequencies of two modes and allows enhancing the sensitivity [2–4]. These designs often utilize the design of flexural beams or tuning electronics to adjust the structural frequency for the purpose of mode-matching, which could increase the sensitivity of the system. However, the high sensitivity of these mode-matched gyroscopes is always at the cost of bandwidth or robustness and is susceptible to structural and environmental parameter variations. Meanwhile, the structural coupling between two modes is also a drawback.

To resolve the problems in the single-DOF MEMS vibratory gyroscopes, some multi-DOFmicro machined vibratory gyroscopes have been presented by increasing DOF of the drive-mode or sense-mode. For the purpose of improving the robustness of MEMS vibratory gyroscopes, the 2-DOFvibratory structure has been utilized in drive-mode or sense-mode to increase the bandwidth of the respective mode. A 3-DOF MEMS vibratory gyroscope with 2-DOF drive-mode and 1-DOF sense-mode is described [5], which uses 2-DOF dynamic vibration absorber(DVA) structure in drive-mode. This design illustrated the wide bandwidth of drive-mode, but there is a narrow bandwidth of sense-mode. Another type of 3-DOF micro machined vibratory gyroscope, with 1-DOF drive-mode and 2-DOF DVA structure in sense-mode, is described [6], which showed the wide bandwidth of sense-mode.

However, there is the limitation of structural design space in the DVA architecture. Moreover, the tradeoffs among operational frequency, bandwidth and die size, as well as detection capacitance must be considered [7]. To overcome the limitation in the DVA structure, a complete 2-DOF architecture with two masses and three springs has been employed in the sense-mode of some 3-DOF micro machined vibratory gyroscopes [8–10]. This design demonstrated the advantage of gain and bandwidth in sense-mode. However, these designs merely improve the bandwidth of drive-mode or sense-mode, respectively, and in the other mode, the robustness also requires improvement.

Recently, a MEMS gyroscope with 2-DOF drive-mode and sense-mode has been proposed to improve the robustness of both drive-mode and sense-mode [11], which utilizes the complete 2-DOF vibratory structure to provide a robust gain-bandwidth frequency region and inherently improves the robustness of drive-mode and sense-mode at the same time. The device decouples drive-mode and sense-mode by adding decoupled mass and proof mass and, concurrently, a complete 2-DOF structure is formed in both modes. Compared to the previous 1-DOF structure, one of the advantages of utilizing the complete2-DOF structure is its robust gain-bandwidth frequency region. Meanwhile, the implementation of the complete 2-DOF system will reduce the complexity of electronic circuits and eliminate the electronic noise due to the complex control circuit in drive-mode [12,13]. However, the gain of sense-mode also needs to increase in order to improve the sensitivity of the device. In this paper, another novel 4-DOFMEMS vibratory gyroscope structure is proposed. Based on this 4-DOF MEMS vibratory gyroscope structure, a novel gyroscope array structure is presented. The proposed gyroscope array is different from the previous gyroscope structure, which combines several gyroscope units into a single gyroscope structure in a special way. The proposed gyroscope array could improve the gain of sense-mode compared to the previous 4-DOF gyroscope [11].

The rest of this paper is organized as follows. Section 2 introduces the design of 4-DOF gyroscope unit. Section 3 presents the structural design of the proposed gyroscope array based on the proposed gyroscope unit. Section 4 gives a theoretical dynamics analysis and the solutions of the gyroscope array. Section 5 reports the comparative simulation results, and Section 6 concludes the paper with a summary of results.

## 2. Gyroscope Unit

**Figure 1.**
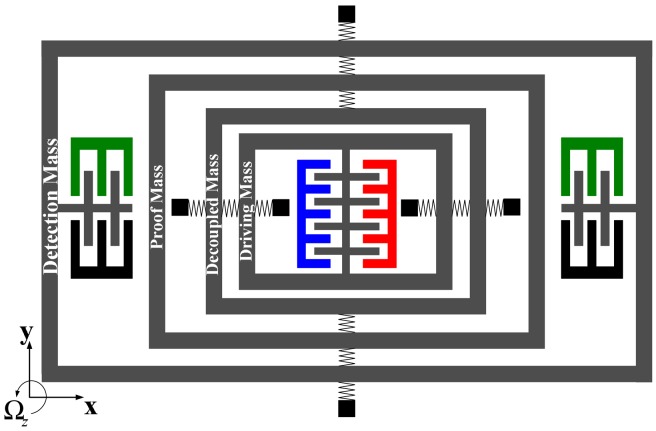
The schematic of proposed gyroscope unit.

The conceptual schematic of proposed gyroscope unit is shown in Figure 1. Similar to the multi-DOF gyroscope [11], the complete 2-DOF vibratory structure is implemented in both drive-mode and sense-mode, which provides both modes with a large bandwidth. The drive-combs are set on the driving mass to drive drive-mode to oscillate along with *x* axis. However, unlike the previous 4-DOF MEMS vibratory gyroscope [11], the elements of drive-mode are inside the schematic and the sense-mode is around the drive-mode. The sensing-capacitors are set on the detection mass used to sense the Coriolis-induced changing capacitance. The detection mass is set at the edge of the schematic, which will be suitable for combining several gyroscope units into a gyroscope array.

**Figure 2.**
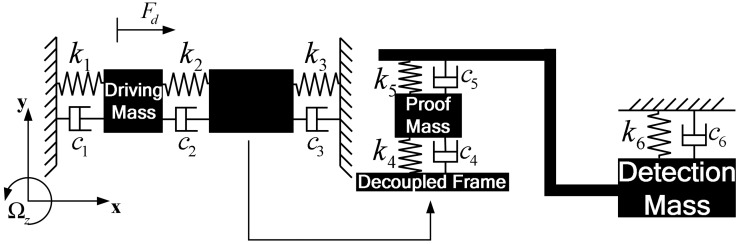
Model of the proposed 4-DOF MEMS vibratory gyroscope.

The lump model of proposed 4-DOF MEMS gyroscope is simplified, as shown in Figure 2.The proposed gyroscope consists of driving mass, decoupled frame, proof mass and detection mass and springs. The driving mass, decoupled frame and springs, *k*_1_, *k*_2_, *k*_3_, form the complete 2-DOFdrive-mode and the proof mass, detection mass and springs, *k*_4_, *k*_5_*, k*_6_, form the complete 2-DOFsense-mode. Drive-mode and sense-mode are linked to each other through decoupled frame and proof mass, which isolate vibration between both modes.

When the gyroscope unit is operated, the driving force is applied to driving mass and drives driving mass and decoupled frame to vibrate in drive direction (*x*). Moreover, proof mass could oscillate with decoupled frame in drive direction (*x*). If an angular rate Ω*_z_* is input along the direction (*z*) perpendicular to *x* – *y* plane, the Coriolis-induced force will excite proof mass and detection mass to move in sense direction (*y*). Then the capacitors on detection mass will change, which helps pick up the motion and calculate the input angular rate Ω*_z_*.

## 3. Gyroscope Array

**Figure 3.**
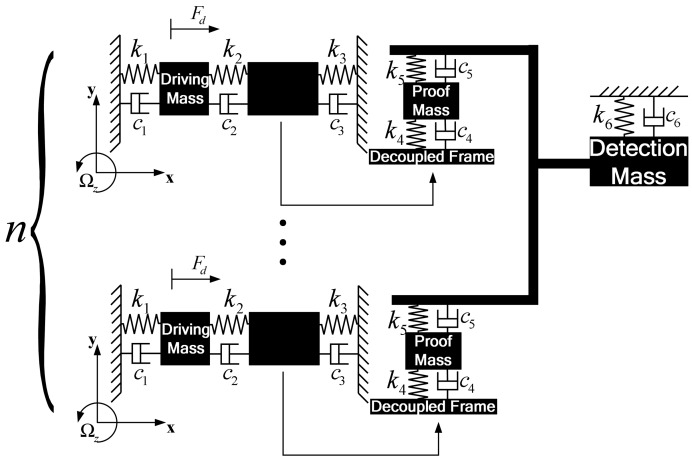
Lump model of the 4-DOF MEMS vibratory gyroscope array.

In this section, a gyroscope array will be designed based on the gyroscope unit presented in Section 2.The lump model of gyroscope array is shown in Figure 3. There are a group of gyroscope units forming a gyroscope array. All the units utilize the identical elements of drive-mode and proof mass respectively, but there is just a detection mass, which is utilized by all the gyroscope units. The proof masses of every gyroscope unit are connected to the unique detection mass through a beam.

**Figure 4.**
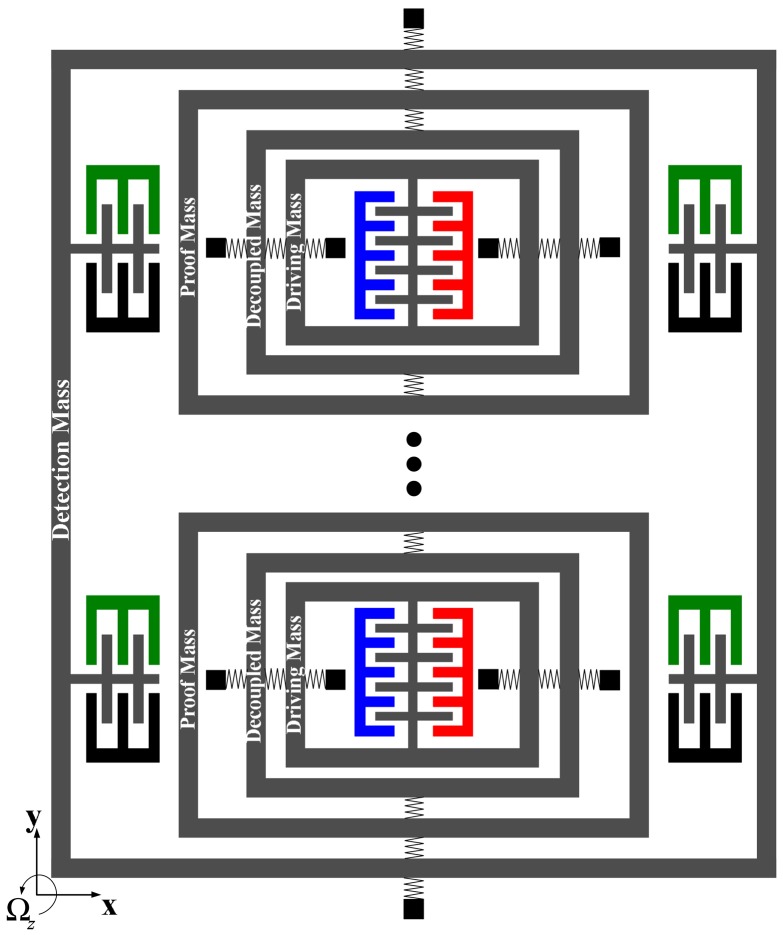
Combination of 4-DOF MEMS vibratory gyroscopes.

In the design of gyroscope array, similar to gyroscope unit, the complete 2-DOF vibratory structureis implemented in drive-mode of every gyroscope unit. In addition, the complete 2-DOF drive-mode could ensure the desired bandwidth the same as a gyroscope unit. In sense-mode, however, the complete2-DOF sense-mode of a gyroscope unit is changed into another model as shown in Figures 3 and 4.The proof masses also belong to the respective gyroscope unit, but the detection masses of all the units in gyroscope array are combined to a common detection mass, which is used by all the units. Then the sense-mode of every gyroscope unit is also a complete 2-DOF vibratory structure providing a wide bandwidth to sense-mode of gyroscope array.

When the gyroscope array is operated, the driving masses of all the gyroscope units are driven along *x* axis by the sinusoidal forces with the same frequency, amplitude and phase, which ensure that the entire drive-mode will work synchronously. When there is an angular rate Ω*_z_* perpendicular to the *x* – *y* plane, the Coriolis-induced force is applied to the respective proof mass. Then all the proof masses are driven to oscillate along *y* axis and detection mass is excited by the force transmitted from all the proof masses. The Coriolis-induced motion is picked up by the detection mass, which can help calculate the input angular rate Ω*_z_*.

Compared with a single gyroscope unit, the gyroscope array combines several (*i.e.*, *n*) gyroscope units and possesses a unique detection mass, which will enlarge the Coriolis-induced force applied to the detection mass and increase the amplitude of the detection mass. The enlarged amplitude of the detection mass will increase the changing capacitance and sensitivity of the device. As shown in Figures 3 and 4, there are several independent drive-mode elements and proof masses, which form a special drive-mode and could improve insensitivity to structural parameters variations. The detailed analysis will be given later.

## 4. Theoretical Analysis

### 4.1. Drive-Mode

As the structural schematic of the micro machined vibratory gyroscope is shown in Figures 2 and 3,all the gyroscope units use the identical drive-mode elements and the dynamic equations of drive-mode can be represented by simplified equations,

(1) $$m_{1}{\ddot{x}}_{1}+(c_{1}+c_{2}){\dot{x}}_{1}-c_{2}{\dot{x}}_{2}+(k_{1}+k_{2})x_{1}-k_{2}x_{2}=F_{d}$$

(2) $$(m_{f}+m_{2}){\ddot{x}}_{2}-c_{2}{\dot{x}}_{1}+(c_{2}+c_{3}){\dot{x}}_{2}-k_{2}x_{1}+(k_{2}+k_{3})x_{2}=0$$

where *x*_1_ and *x*_2_ represent the motion of driving mass *m*_1_ and decoupled frame *m_f_* (with proof mass *m*_2_)along with *x* axis. The driving force *F_d_* is sinusoidal applied to the driving mass and drives driving mass*m*_1_ and decoupled frame *m_f_* (with proof mass *m*_2_) to oscillate in the drive direction(*x*). The proof mass*m*_2_ provides the link between two modes and without input angular rate, it only oscillates in the drive direction (*x*) with the decoupled frame *m_f_*. When there is rotation about the *z* axis perpendicular to the *x* – *y* plane, the Coriolis-induced force is applied to the proof mass *m*_2_ and drives proof mass *m*_2_ and detection mass *m*_3_ to move in the sense direction(*y*).

Because the decoupled frame *m_f_* and proof mass *m*_2_ provide the link between two vibratory modes and their vibration along *x* axis will be transferred to *y* axis due to Coriolis effect, the dynamic response(*x*_2_) of decoupled frame *m_f_* (with proof mass *m*_2_) along *x* axis will occur. Assuming the solution *x*_2_ are sinusoidal, according to Laplace transform the steady solution for *x*_2_ is given by,

(3) $$x_{2}=\frac{(k_{2}+j\omega c_{2})F_{d}}{\Delta_{d}(\omega )}$$

where Δ*_d_*(*ω*) = (*k*_1_ + *k*_2_ – *m*_1_*ω*^2^ + *jω*(*c*_1_ + *c*_2_))(*k*_3_ + *k*_2_ – (*m_f_* + *m*_2_)*ω*^2^ + *jω*(*c*_2_ + *c*_3_)) – (*k*_2_+ *jωc*_2_)^2^.The Equation (2) provides theoretical argument for performance analysis. The structural frequency of the complete 2-DOF dynamic system in drive-mode can be defined by,

(4) $$\omega_{d1}^{2}=\frac{k_{1}+k_{2}}{m_{1}},\omega_{d2}^{2}\frac{k_{2}+k_{3}}{m_{f}+m_{2}}$$

according to vibration dynamics, the anti-resonant frequency *ω_d_*_0_ of the driving mass *m*_1_ is always between the two resonant frequencies of drive-mode. For obtaining the precise position of the robust operational region in drive-mode, the structural frequencies *ω_d_*_1_ and *ω_d_*_2_ are set equal to *ω_d_*_0_.

Assuming zero-damping system and solving the eigenvalue equation Δ*_d_*(*ω*) = 0, the resonant frequencies of drive-mode can be obtained,

(5) $$\omega_{dH,L}=\sqrt{\omega_{d0}^{2}\pm \sqrt{\frac{k_{2}^{2}}{m_{1}(m_{2}+m_{f})}}}$$

where *ω_dH,L_* are the high and low resonant frequencies of drive-mode. Define the resonant peak spacing Δ*_d_* as,

(6) $$\Delta_{d}=\omega_{dH}-\omega_{dL}$$

And by using Equations (4), (5) and (6), the design equations of the stiffnesses in drive-mode are given by,

(7) $$\{\begin{array}{l} k_{2}=\Delta_{d}\sqrt{m_{1}(m_{2}+m_{f})}\sqrt{\omega_{d0}^{2}-0.25\Delta_{d}^{2}}\\ k_{1}=m_{1}\omega_{d0}^{2}-k_{2}\\ k_{3}=(m_{f}+m_{2})\omega_{d0}^{2}-k_{2} \end{array}$$

It is obvious that the resonant peak spacing Δ*_d_* of drive-mode can be set independently of the operational frequency *ω_d_*_0_*,* which eliminates the limitation of DVA structure [7]. After the layout ofdriving mass *m*_1_ and decoupled frame *m_f_* (with proof mass *m*_2_), the operational frequency can be set by adjusting the stiffnesses. In the proposed gyroscope array, its drive-mode includes several independent same driving elements belonging to respective gyroscope units and their dynamics equations and design equations are the same as Equations (1), (2) and (7), which is a different point in contrast to others.

### 4.2. Sense-Mode

As is shown in Figures 2 and 3, sense-mode elements of the gyroscope unit and gyroscope array are different from each other. Because there is proof mass *m*_2_ in sense-mode of all gyroscope units and they are identical, the motion of proof mass *m*_2_ in sense-mode of every gyroscope unit can be described by the same equation,

(8) $$m_{2}{\ddot{y}}_{1}+(c_{4}+c_{5}){\dot{y}}_{1}-c_{5}{\dot{y}}_{2}+(k_{4}+k_{5})y_{1}-k_{5}y_{2}=F_{C}$$

where *y*_1_ represents the coordinate of proof mass *m*_2_ in all gyroscope units and *y*_2_ represents the coordinate of detection mass *m*_3_ in gyroscope array. *F_C_* is the Coriolis-induced force applied to proof mass *m*_2_. In gyroscope array, the unique detection mass *m*_3_ is utilized by all the gyroscope units of gyroscope array and its motion can be given by,

(9) $$m_{3}{\ddot{y}}_{2}-nc_{5}{\dot{y}}_{1}+(nc_{5}+c_{6}){\dot{y}}_{2}-nk_{5}{\dot{y}}_{1}+(nk_{5}+k_{6})y_{2}=0$$

where *n* is the number of gyroscope units in proposed gyroscope array.

Because the detection mass *m*_3_ is used to pick up the Coriolis-induced motion through the sensing-capacitor in it, the motion *y*_2_ of detection mass *m*_3_ is mainly focused on. Using Equations (8) and (9), the solution of the detection mass can be obtained,

(10) $$y_{2}=\frac{n(k_{5}+j\omega c_{5})F_{C}}{\Delta_{s}(\omega )}$$

where Δ*_s_*(*ω*) = (*k*_4_ + *k*_5_*- m*_2_*ω*^2^ + *jω*(*c*_4_ + *c*_5_))(*nk*_5_ + *k*_6_ – *m*_3_*ω*^2^ + *jω*(*nc*_5_ + *c*_6_)) - *n*(*k*_5_ + *jωc*_5_)^2^.

After the design and layout of the masses, the stiffnesses of all the springs must be calculated. According to vibration dynamics, the anti-resonant frequency *ω_s_*_0_ of the proof mass *m*_2_ is always between the two resonant frequencies of sense-mode. Define the structural frequencies of sense-mode by,

(11) $$\omega_{s1}^{2}=\frac{k_{4}+k_{5}}{m_{2}},\omega_{s2}^{2}=\frac{nk_{5}+k_{6}}{m_{3}}$$

For obtaining the precise position of the robust operational region in sense-mode, we set*ω_s_*_1_ = *ω_S_*_2_ = *ω_s_*_0_ (*ω_s_*_0_ is the anti-resonant frequency of proof mass). Then assuming zero-damping system and solving the eigenvalue equation (Δ*_s_*(*ω*) = 0) of sense-mode, the resonant frequencies of sense-mode can be obtained,

(12) $$\omega_{sH,L}=\sqrt{\omega_{s0}^{2}\pm \sqrt{\frac{nk_{5}^{2}}{m_{2}m_{3}}}}$$

where *ω_sH,L_* are the high and low resonant frequencies of sense-mode. The resonant peak spacing Δ*_s_* of sense-mode are defined by,

(13) $$\Delta_{s}=\omega_{sH}-\omega_{sL}$$

Using Equations (11), (12) and (13), the desired stiffnesses *k*_4_, *k*_5_*, k*_6_*,* can be given by,

(14) $$\{\begin{array}{l} k_{5}=\frac{1}{\sqrt{n}}\Delta_{s}\sqrt{m_{2}m_{3}}\sqrt{\omega_{s0}^{2}-0.25\Delta_{s}^{2}}\\ k_{4}=m_{2}\omega_{s0}^{2}-k_{5}\\ k_{6}=m_{3}\omega_{s0}^{2}-{\text{nk}}_{5} \end{array}$$

which are the design equations of sense-mode in gyroscope array. The design equations of sense-modeare different from those of drive-mode due to the different structures of both modes. Compared with other gyroscope structures, another different point is that it utilizes several proof masses to enlarge the Coriolis force and their dynamics equations are the same as Equation (8).

Both Equations (7) and (14) are the design equations of proposed gyroscope array and the stiffnesses*k*_1_*, k*_2_*, k*_3_*, k*_4_, *k*_5_*, k*_6_*,* can be gained in terms of the design equations. Because the resonant peak spacing Δ*_d_*_0_ and Δ*_s_*_0_ determine the bandwidths of drive-mode and sense-mode, and the robust region is up to the central operational frequency, Δ*_d_*_0_ and Δ*_s_*_0_ should be set appropriately. Meanwhile, the anti-resonant frequencies *ω_d_*_0_ equal to *ω_s_*_0_ is the central operational frequency. Then the bandwidth of drive-mode will be highly matched with that of sense-mode, which provides the desired bandwidth of the system. At the same time, the number *n* of gyroscope units in the gyroscope array should be selected appropriately to ensure that the combination of gyroscope units increases the sensitivity of the system without decreasing the desired bandwidth of the system.

## 5. Simulation Results

According to the theoretical analysis above, the solutions of both modes and design equations of the system are given by Equations (3), (7), (10) and (14). The values of the main parameters in the gyroscope unit and proposed gyroscope array are given in Table 1. The stiffness could be calculated by the design equations. Then the simulation will be shown in terms of these analysis.

**Figure 5.**
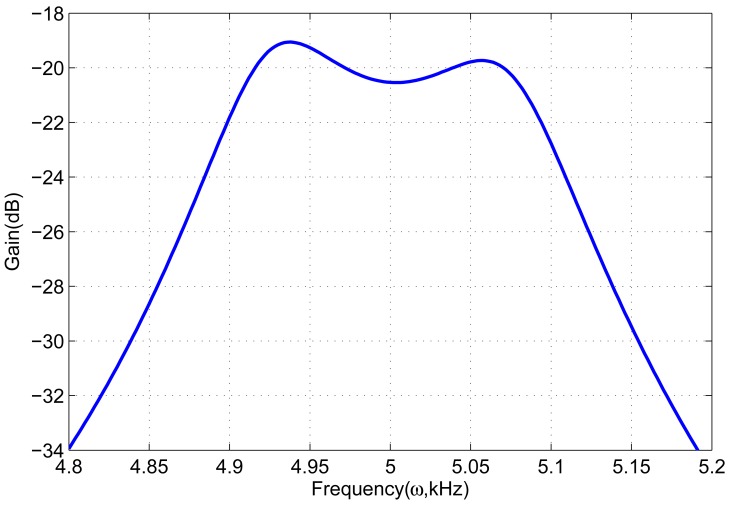
Frequency response of drive-mode.

Since the drive-modes of both gyroscope unit and gyroscope array are identical, their amplitude-frequency responses are the same. Figure 5 shows the frequency response of drive-mode. It clear illustrates a 3 dB bandwidth of about 190 Hz around the central operational frequency 5 kHz. Thus, the gyroscope unit and gyroscope array have the same bandwidth and gain in the drive-mode.

**Figure 6.**
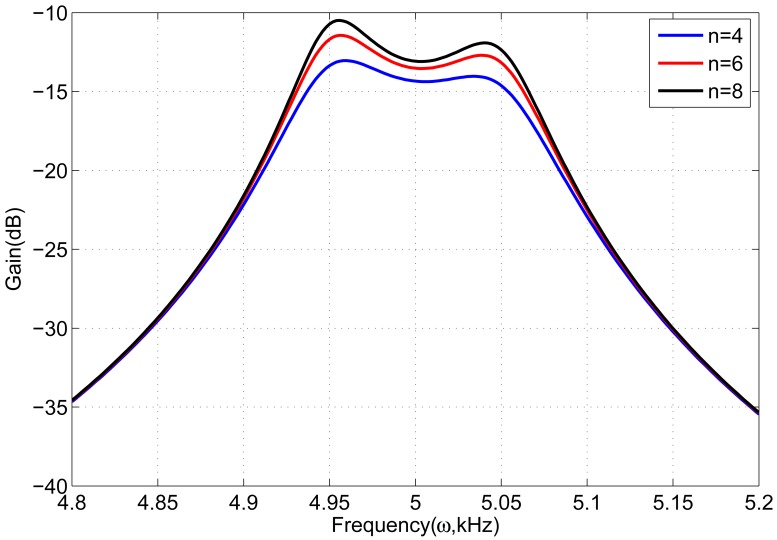
Frequency response of gyroscope array with different number of gyroscope unitsin sense-mode.

**Figure 7.**
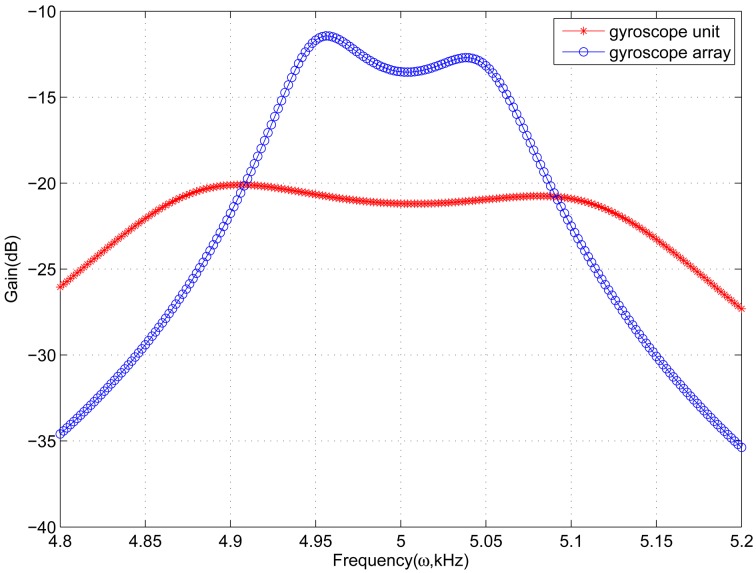
Frequency response of gyroscope unit and gyroscope array(*n* = 6) in sense-mode.

In sense-mode of the gyroscope array, the number *n* of gyroscope units needs to be set. Because the number *n* has an effect on the frequency response of sense-mode, it must be set appropriately to satisfy the desired bandwidth and gain. Figure 6 shows frequency response of sense-mode with different *n.* When the number is 6, the frequency response has a high gain and also changes softly, which is better than others. Figure 7 demonstrates the frequency response of gyroscope unit and gyroscope array in sense-mode. In gyroscope unit, the 3 dB bandwidth is about 300 Hz around the central operational frequency 5 kHz, and the gain is from −23 dB to −20 dB; in gyroscope array, the 3 dB bandwidth is about100 Hz around the central operational frequency 5 kHz, and the gain is increased by 8 dB, from −15 dBto −12 dB.

Through combining gyroscope units into a gyroscope array, the gain of sense-mode is increased andthe sensitivity is improved inherently. On the other hand, the value of detection mass is also increased by 6 times, as compared to the gyroscope unit shown in Table 1. Therefore, the changing capacitance is6 times that of the gyroscope unit, which increases the output signal and also improves the sensitivity. Although the bandwidth of gyroscope array is decreased to 100 Hz, it satisfies the need in practical applications. Meanwhile, the simulation results show that the bandwidth and central frequency of drive-mode are highly matched with those of sense-mode, which provides the system with a bandwidth of 100 Hz and improves the inherent robustness of proposed gyroscope array.

There is some tolerance in practical fabrication and the springs are susceptible to structural parameter variations. The key spring *k*_5_ will be considered because the springs *k*_5_ provide the link between detection mass, and all the proof masses of gyroscope units in the proposed gyroscope array as shown in Figure 3. Therefore, we mainly discuss the effect of *k*_5_ on the frequency response. The desired value of *k*_5_ can be calculated by design Equation (14).

**Figure 8.**
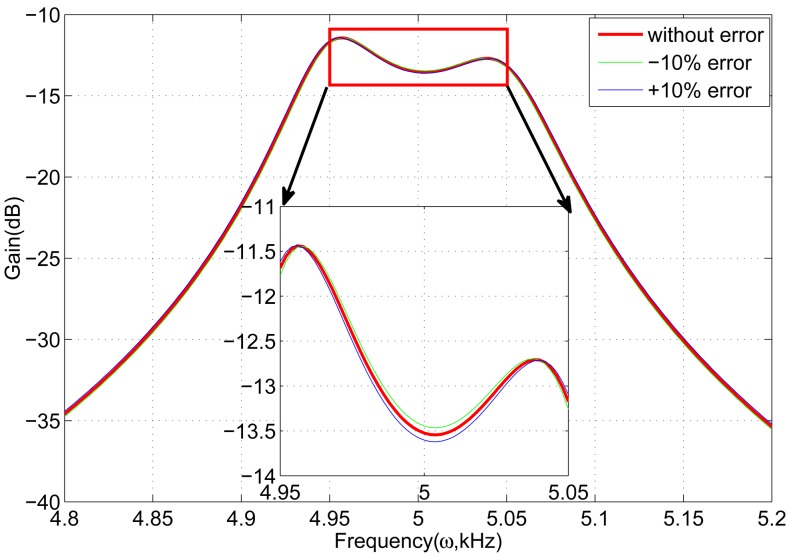
Effect of one of springs *k*_5_ on frequency response.

**Figure 9.**
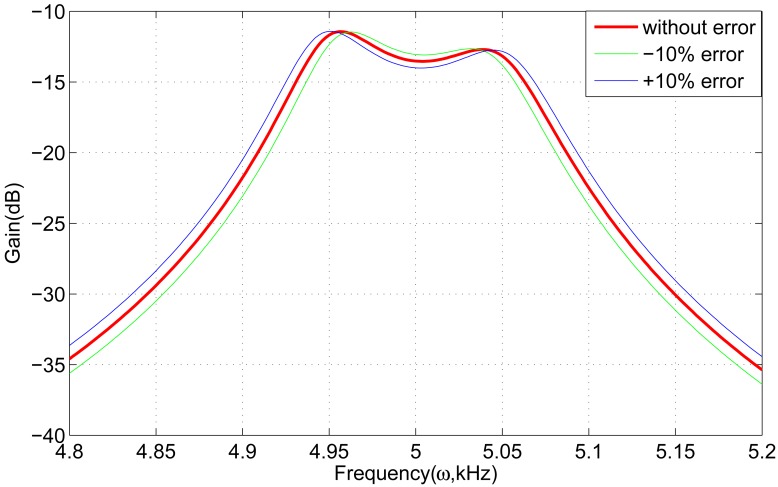
Effect of all the springs *k*_5_ on frequency response.

First, if the spring *k*_5_ in one of the gyroscope units changes with ±10%, the frequency response is shown in Figure 8. It is clear that the curves have nearly no fluctuation, which demonstrates that the gyroscope array is insensitive to one changing spring *k*_5_. Subsequently, all the springs *k*_5_ are considered. Assuming that the sum of all the springs *k*_5_ changes with ±10%, the frequency response is shown in Figure 9. The frequency response curves change within 0.5 dB compared to the curve without error.

## 6. Conclusion

A novel 4-DOF MEMS vibratory gyroscope unit is proposed first. The complete 2-DOF vibratory structure is utilized in both drive-mode and sense-mode, which provides the desired bandwidth of the system and improves the inherent robustness. Unlike the previous 4-DOF MEMS vibratory gyroscope, the elements of drive-mode lay inside the schematic of the gyroscope unit and the elements of sense-mode are around drive-mode, which is for the convenience of combining some gyroscope units.

Based on the proposed gyroscope unit, a gyroscope array is designed to improve the sensitivity of the system. In gyroscope array, all the gyroscope units use the identical 2-DOF vibratory structure and there is also a proof mass in all gyroscope units. The unique detection mass is shared by all the gyroscope units so that the sensitivity of gyroscopes will be improved and the bandwidth is also adequate.

Compared to the gyroscope unit, the gain of frequency response in sense-mode of the gyroscope array(*n* = 6) is increased by 8 dB and the changing capacitance is increased by six times, which improve the sensitivity inherently. Furthermore, the bandwidth and central frequency are highly matched with each other in both modes, which provides the system with inherent robustness. Although the bandwidth is decreased to 100 Hz, it could satisfy the need in practical applications.

## Figures and Tables

**Table 1.** Parameters of the gyroscope unit and proposed gyroscope array

**Parameters**	**Values of gyroscope unit**	**Values of gyroscope array**
*m*_1_	2.36*e*^−7^ kg	2.36*e*^−7^ kg
*m*_1_	5.23*e*^−8^ kg	5.23*e*^−8^ kg
*m*_2_	2.547*e*^−7^ kg	2.547*e*^−7^ kg
*m*_3_	1.35*e*^−7^ kg	8.1*e*^−7^ kg
*ω_s_*_0_ = *ω_d_*_0_	5.0 kHz	5.0 kHz
Δ*_d_*	150 Hz	150 Hz
Δ*_s_*	250 Hz	100 Hz
*C*_1_	l*e*^−4^ Ns/m	l*e*^−4^ Ns/m
*c*_2_	5*e*^−6^ Ns/m	5*e*^−6^ Ns/m
*c*_3_	2*e*^−4^ Ns/m	2*e*^−4^ Ns/m
*c*_4_	l*e*^−4^ Ns/m	l*e*^−4^ Ns/m
*c*_5_	5*e*^−6^ Ns/m	5*e*^−6^ Ns/m
*c*_6_	2*e*^−4^ Ns/m	2*e*^−4^ Ns/m
